# Effects of Dynamic Stretching Combined with Manual Therapy on Pain, ROM, Function, and Quality of Life of Adhesive Capsulitis

**DOI:** 10.3390/healthcare12010045

**Published:** 2023-12-24

**Authors:** Jeong-Min Choi, Eun-Young Cho, Byoung-Hee Lee

**Affiliations:** 1 Graduate School of Physical Therapy, Sahmyook University, Seoul 01795, Republic of Korea; thecubedphysio@gmail.com; 2 Institutional Research Center, Sahmyook University, Seoul 01795, Republic of Korea; myjesus3535@syu.ac.kr; 3 Department of Physical Therapy, Sahmyook University, Seoul 01795, Republic of Korea

**Keywords:** adhesive capsulitis, dynamic stretching, static stretching, quality of life

## Abstract

This study was conducted to evaluate the effects of dynamic stretching combined with manual therapy on pain, range of motion, function, and quality of life in patients with adhesive capsulitis. The participants were randomly divided into two groups: the dynamic stretching combined with manual therapy (DSMT) group (*n* = 17) and the static stretching combined with manual therapy (SSMT) group (*n* = 17). Both groups received manual therapy for 10 min and two sessions per week for 4 weeks. The DSMT group also performed additional dynamic stretching for 20 min per session, two sessions per week for 4 weeks. The SSMT group practiced additional static stretching for 20 min per session, two sessions per week for 4 weeks. The pain, ROM, function, and quality of life were measured and evaluated before and after treatment. There were significant improvements in the outcomes of pain, flexion and abduction of shoulder ROM, Shoulder Pain and Disability Index (SPADI), and the physical component score and mental component score of the Short Form-36 (SF-36) in both groups. Additionally, the external and internal rotation of the shoulder ROM and the SF-36 general health factor increased significantly more in the A group (DSMT group) compared to the B group (SSMT). In conclusion, dynamic stretching plus manual therapy offers the same results as static stretching plus manual therapy, but with additional improvement in internal and external rotation.

## 1. Introduction

Degenerative changes around the shoulder joint can cause pain and limit the range of motion, resulting in a condition known as frozen shoulder or adhesive capsulitis [1]. Adhesive capsulitis is classified into two types: primary and secondary. Primary idiopathic adhesive capsulitis is characterized by the onset of pain and progressive stiffness of the glenohumeral joint junction without a specific cause [2]. Secondary factors have been subdivided into systematic, intrinsic, and extrinsic factors according to their occurrence in several review papers [3]. Systemic factors include diabetes and hypothyroidism, while extrinsic factors include rotator cuff pathology, cervical disc disease, stroke, Parkinson’s disease, and humerus fractures [4]. External factors can directly or indirectly impact the shoulder joint and surrounding tissues, potentially leading to restricted shoulder mobility and the onset of pain. Cervical disc disease refers to the damage of discs in the cervical region. Such damage can exert pressure on nerves and surrounding tissues, potentially causing pain or abnormal sensations. In this scenario, it can affect the nerves and tissues between the shoulder and neck, thereby limiting the mobility of the shoulder joint.

A stroke refers to a condition where there is a sudden blockage of blood flow or a rupture of blood vessels in the brain, resulting in damage to brain tissues. Stroke-induced damage to the nervous system can occur, potentially leading to restricted movement of the shoulder joint. Parkinson’s disease is a central nervous system disorder that affects motor functions. This disease disrupts muscle control and movement, which can consequently restrict the motion of the shoulder. The humerus is the bone in the arm that connects the shoulder joint to the forearm. Fractures in the humerus can refer to partial or complete breaks in the bone, which can impact the surrounding tissues. Following a fracture, adhesive capsulitis may occur, particularly during the recovery process.

Physical therapists have access to various non-surgical intervention methods for adhesive capsulitis, including electrical therapy, joint mobilization, stretching, manual therapy, and patient education [3,4]. Adhesive capsulitis causes pain and joint motion limitation that intensify about 9 to 12 weeks after onset. This is followed by a gradual decrease in pain over the next 9 to 12 weeks, leaving only joint motion limitation. The restriction of joint motion is then gradually restored over the last 9 to 12 weeks. To improve the range of motion, stretching techniques should be applied within the range of limited movement. Stretching is a therapeutic technique that enhances muscle extensibility and increases joint mobility.

Myofascial Release Technique-based manual therapy has found application in the rehabilitation of various musculoskeletal injuries, such as neck pain, low back pain, scapulohumeral periarthritis, and functional ankle instability. The Myofascial Release Technique is a manual therapy approach that focuses on the manipulation of the body’s fascial system to alleviate musculoskeletal pain and dysfunction. This technique involves applying gentle, sustained pressure to the myofascial connective tissues, which encompass the muscles and the fascia (a type of connective tissue that surrounds and supports muscles). By releasing tension and restrictions within these tissues, MFR aims to restore mobility, improve posture, and alleviate pain associated with various musculoskeletal conditions. This approach is often integrated into rehabilitation programs for injuries such as neck pain, lower back pain, scapulohumeral periarthritis, and functional ankle instability. Clinical applications and associated experiments with the Myofascial Release Technique have shown a growing trend. The current study has demonstrated that the Myofascial Release Technique effectively reduces fibrous adhesion, optimizes fascial slip, and provides relief for both acute and chronic conditions [5,6]. As this technique directly targets the human fascia, it has a significant impact on regulating deep muscles and connective tissues, restoring fascial tension, and positively affecting pain relief and function improvement [7,8]. Furthermore, the Myofascial Release Technique facilitates the release and extension of soft tissues, enhances local blood circulation, and restores the range of motion in restricted joints. This, in turn, helps alleviate muscle pain, reduce stiffness, and mitigate excessive fatigue to a certain extent [9,10]. Myofascial release is a manual therapy technique that focuses on releasing tension and tightness in the myofascial tissues, which are the thin, strong connective tissues in muscles such as the upper trapezius, pectoralis major, pectoralis minor, rhomboid, and middle trapezius. Myofascial release-based manual therapy is thought to have the potential to alleviate chronic pain conditions, such as lower back pain, neck pain, and headaches, by releasing tension and trigger points in the fascia, thereby reducing muscle pain and discomfort [11].

Stretching is a method for consciously stretching muscles or ligaments and continuing elongation for a certain time and is described as a general flexibility exercise, stretching exercise, and mobility exercise. All muscles are accepted as specific expressions that increase their joint mobility by stretching [12].

Stretching is divided into static stretching, carried out at the end of the range of motion without recoil, and dynamic stretching, carried out to the end of the range of motion while giving rebound within the range of motion. Static stretching involves stretching the muscles to their maximum length, and when tension decreases through stretching, muscle length can increase. However, this can seriously damage muscle function [13]. In other words, excessive or aggressive static stretching, which involves stretching muscles to their maximum length, can potentially lead to decreased muscle strength and power, as well as the risk of micro-tears in muscle tissue, highlighting the importance of incorporating a balanced approach to stretching for overall muscle health and function. Static stretching does not involve rapid limb movement or voluntary muscle contractions. The force applied during static stretching is generated by external factors such as gravity, tools, or physical therapists, unlike dynamic stretching. This force should be applied slowly and continuously, and maintaining regular breathing during static stretching is important [14].

Dynamic stretching, on the other hand, is a stretching exercise that involves controlled movements through specific actions. It promotes dynamic flexibility, replicates movement patterns necessary during specific actions, including multiple joints, and enhances coordination during movement, known as neuromuscular training [14]. Some studies comparing dynamic stretching to static stretching have shown that, in healthy subjects, the improvement in the range of motion (ROM) is similar to or even greater with dynamic stretching, particularly in acute effects, and it is important to note that these studies do not specifically pertain to the shoulder joint [15,16]. Additionally, it is important to note that in studies conducted on healthy subjects and not specific to the shoulder joint, dynamic stretching has been shown to improve the range of motion (ROM) less than static stretching, which demonstrates the opposite of what was originally stated [17].

One perceived benefit of dynamic stretching is that it does not lead to subsequent performance impairments and may even enhance performance [18]. Furthermore, the effects of dynamic movements are influenced by the activation of neuromuscular mechanisms such as muscle spindle reflex activity, increased corticospinal activity, strengthening of sustained inward currents (amplification of motor output), and an increase in enzymatic cycling due to muscle contractions. These mechanisms can induce a rise in muscle temperature, leading to improved muscle coordination and enhanced proprioceptive sensitivity [19].

However, neither dynamic nor static stretching has been studied as an effective intervention for patients with adhesive capsulitis. Although stretching is effective for patients, it has not yet been studied how it can be applied as an effective interventional treatment method for patients with adhesive capsulitis by comparing dynamic stretching with static stretching.

Patients with adhesive capsulitis experience a low quality of life due to their passive and active shoulder mobility limitations [20]. Health-related quality of life is a concept that encompasses subjective satisfaction with physical, psychological, economic, spiritual, and social functions in an individual’s daily life, representing an individual’s physical and mental health status well [21]. The World Health Organization, currently gaining the most widespread support as a health concept, emphasizes that physical, mental, and social dimensions should be reflected in the scale when measuring the level of health [22]. Due to the comprehensive nature of the quality of life concept, the explanatory model includes related factors of various dimensions. It is necessary to consider not only the physical health but also the mental health of patients with adhesive arthritis.

Therefore, in this study, dynamic and static stretching techniques were applied to patients with adhesive capsulitis to examine their effects on pain, range of motion, function, and quality of life, and the basis for effective rehabilitation interventions for the treatment of adhesive capsulitis. We would like to present it as a material. In addition, we will examine how the therapist’s rehabilitation intervention method affects not only the physical change in the patient but also the psychological change and quality of life. In other words, the purpose of the study was to provide data on treatment options for patients suffering from adhesive capsulitis of the shoulder through the evaluation of dynamic and static stretching on pain, range of motion, function, and quality of life.

## 2. Materials and Methods

### 2.1. Subjects

This study recruited 39 patients diagnosed with adhesive capsulitis and admitted to a B hospital in Seongnam City, Gyeonggi-do. The study participants were recruited from patients who visited the hospital of the principal investigator. The recruitment method was conducted through a recruitment notice posted on the hospital bulletin board.

The inclusion selection criteria for this study included individuals in their 40s to 60s who experienced reduced shoulder range of motion, with a Numerical Pain Rating Scale score of 4 or higher and persistent pain for more than four weeks. Participants were identified as having adhesive capsulitis, characterized by typical features such as less than 50% restriction in range of motion when compared to the unaffected shoulder’s external and internal rotation. Exclusion criteria consisted of congenital problems in the injured shoulder joint itself, rotator cuff damage, dislocation or subluxation, fracture, shoulder surgery history within the past 6 months, clear cervical disease, systemic disease, nerve damage, diabetes, thyroid lesions, and infections.

They all signed a consent form after the procedure and purpose of the study were explained. This study was approved by the Sahmyook University Institutional Review Board (approval number: SYU 2022-07-010-001) and the Clinical Research Information Service (KCT0007727). The objectives and procedures used in the study were fully understood by the subjects. This study upheld the ethical principles of the Declaration of Helsinki.

### 2.2. Experimental Procedure

Before recruiting participants for this study, we performed a power analysis using G*Power version 3.1.9.7 (Heinrich-Heine Universität, Düsseldorf, Germany); an overall effect size index of 0.85 was obtained for all the outcome measures, with a probability of 0.05, to minimize type II errors (power of 80%). Because the estimated target sample size was 36, we recruited 39 participants undergoing physical therapy.

In this study, we obtained prior approval to access medical records and confirmed clinical characteristics such as date of onset, cause of onset, and medical history. General characteristics such as gender, age, weight, height, and body mass index (BMI) were also recorded. To minimize bias, therapists were randomly assigned to each group. Among the 39 participants recruited, 34 were included in the study after excluding 5 who were unable to participate in the experiment.

The participants were randomly divided into two groups 1 h before the start of training to minimize errors caused by group selection. All subjects picked up a black or white stone from a box containing 34 pieces of stone that were not visible. White stones were categorized as group A, while black stones were categorized as group B.

The 34 participants were randomly divided into two groups: the dynamic stretching group, which received connective tissue relaxation, manual range of motion exercises, 10 min of manual therapy, and 20 min of dynamic stretching twice a week for 4 weeks; and the static stretching group, which underwent the same protocol with 20 min of static stretching instead of dynamic stretching.

**Figure 1:**
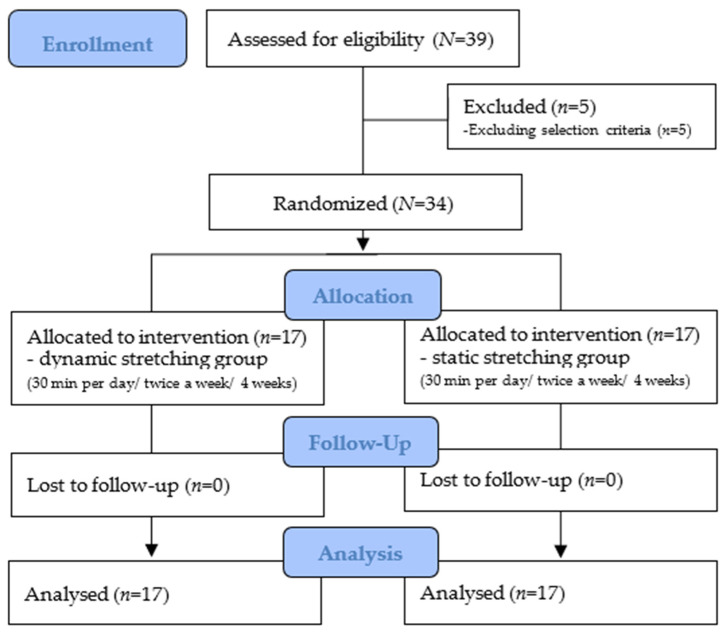
Flow diagram of the overall experimental procedure.

The flow diagram of the overall experimental procedure is shown in Figure 1.

The pre- and post-evaluation items included the range of motion, numeric pain rating scale, shoulder function disability index, and the Korean version of the Short Form-36.

The physical therapists who participated in the study had more than 3 years of experience and received education on adhesive capsulitis, potential problems during the study process, manual therapy, and the study itself. The same therapist applied manual therapy to the patients after practicing the manual therapy beforehand. In this study, dynamic stretching, in addition to manual therapy and static stretching, was implemented to improve pain, range of motion, function, and quality of life in patients with adhesive capsulitis.

Eight physical therapists participated in the study: six therapists provided physical therapy and two therapists provided assessment. Three therapists were assigned to group A, and three therapists were assigned to group B. The participants received treatment from the same therapist twice a week for 4 weeks. Of the two assessors, one administered the NPRS (Numeric Pain Rating Score) and ROM tests, and the other administered the SPADI (Shoulder Pain and Disability Index) and SF-36. Patient treatment and evaluation were conducted separately to ensure the assessors’ blinding to the intervention.

### 2.3. Training Program

#### 2.3.1. Manual Therapy with Dynamic Stretching (Group A)

The manual therapy applied in this study refers to manual therapy based on myofascial release technology. The procedure involved manual tissue relaxation (myofascial release) for shoulder joint-related muscles (upper trapezius, pectoralis major, pectoralis minor, rhomboid, and middle trapezius) for 5 min, followed by 5 min of passive joint range-of-motion exercise. Dynamic stretching techniques were then applied to patients with adhesive capsulitis. The patients performed stretching exercises for specific actions (flexion, abduction, external rotation, and internal rotation motion) while actively engaging in the movements themselves. While performing dynamic stretching, therapists were cautious not to actively engage or tighten the muscles, and they made sure that the shoulders did not become rigid or tense. The order of dynamic stretching was as follows: internal rotation, external rotation, flexion, and external rotation [23]. Patients underwent dynamic stretching while comfortably lying on the treatment table, aiming to perform the stretches up to the maximum range of motion without experiencing pain. After holding the range of motion for 30 s using a recoil-based repetitive exercise, the patient rested for 10 s [24]. Dynamic stretching was performed a total of 20 times, taking approximately 20 min in total, and was continued twice a week for 4 weeks.

#### 2.3.2. Manual Therapy with Static Stretching Application (Group B)

The procedure involved performing myofascial release on the shoulder joint-related muscles (upper trapezius, pectoralis major, pectoralis minor, levator scapulae, and middle trapezius) for 5 min, followed by 5 min of passive joint mobilization. Then, static stretching was applied to patients with adhesive capsulitis. While performing static stretching, therapists were cautious not to actively engage or tighten the muscles, and they made sure that the shoulders did not become rigid or tense. The sequence of static stretching was performed in the following order: internal rotation, external rotation, flexion, and external rotation [23]. The patient lay on the bed in a comfortable position and received passive stretching from the therapist. The therapist moved the patient’s joints to the range without pain. After reaching the end range of motion, the patient held the position for 30 s and then took a 10 s rest [24]. A total of 20 repetitions of static stretching were performed, taking about 20 min. This was performed twice a week for four weeks.

#### 2.3.3. Measurement Tools and Data Collection Process

This study measured pain evaluation using a numerical pain rating scale. The scale ranges from 0 to 10, where 0 indicates no pain and 10 indicates unbearable pain experienced by the patient in the last 24 h. The patients indicated their pain level by marking the average level of pain they were feeling. This simple and highly reproducible method has high sensitivity and reliability, with an intraclass correlation coefficient (ICC) of 0.90 [25].

The range of motion was measured in this study using a goniometer. The shoulder’s range of motion was measured by active range of motion within the pain-free range.

Shoulder flexion assessment is performed with the patient in a supine position with flexed knees. The palm should be facing medially with the thumb up. To start the test, the patient’s arm should be by their side. The axis location is at the middle of the humeral head laterally, the stationary arm should be parallel to the trunk, and the movement arm should be in line with the midline of the humerus (lateral epicondyle). Shoulder abduction assessment is performed with the patient in a supine position with the palm facing upwards and the wrist in supination. To start the test, the arm is to be by the patient’s side. The axis location is at the inferior lateral coracoid process, the stationary arm should be parallel with the trunk, and the movement arm should be in line with the midline of the humerus. Shoulder internal rotation and external rotation assessment are performed with the patient in a supine position with the shoulder abducted to 90 degrees and the length of the humerus on the test side supported on the plinth. The forearm is in a neutral position. The axis location is at the olecranon process of the ulna, the stationary arm should be perpendicular to the floor (vertical), and the movement arm should be in line with the ulnar side of the forearm from the axis point to the ulnar styloid process.

This study measured the function using the Korean version of the Shoulder Pain and Disability Index (SPADI). The Korean version of the SPADI consists of a self-report questionnaire with a total of 13 items divided into 5 items for the pain subscale, 8 items for the function/disability subscale, and 2 subscales. The scoring of each item has the same weight in each area, and the score of each area is converted into a percentage (%), with 0 indicating the perfect condition and 100 indicating the worst condition. The total score is determined by the average score of the 13 evaluation items [26]. The inter-rater test–retest reliability of SPADI was 0.99, and the internal consistency was 0.943 for Cronbach’s alpha [27].

In this study, quality of life was measured using the general health-related quality-of-life measurement tool SF-36 (Short Form-36). The SF-36 was developed as an indicator of overall health status and consists of 36 items and 8 scales. The data values of the 8 scales are converted to obtain a value between 0 and 100, with higher scores indicating a better health status and higher quality of life in the relevant area [22]. The range of test–retest reliability of the Korean SF-36 was *r* = 0.710 to 0.895, and the range of the Cronbach’s Alpha value for internal consistency was 0.930 to 0.938 [28].

### 2.4. Data Analysis

In this study, IBM SPSS ver. 20 (IBM, Chicago, IL, USA) was used for statistical analysis. Descriptive statistics including mean and standard deviation were calculated for the general characteristics of all subjects using the Shapiro–Wilk test for normality verification, and the results showed that they were normally distributed. Descriptive statistics were used to analyze the general characteristics of the participants. Moreover, homogeneity tests were performed before the experiment by the various variables of the two groups. To compare before and after applying dynamic and static stretching, a paired-sample *t*-test was conducted. The interaction effect between groups over time was analyzed using a two-way repeated-measure analysis of variance (ANOVA). The statistical significance level for all data was set at 0.05.

## 3. Results

The subjects of this experiment consisted of a total of 34 individuals, with 17 in the manual therapy group using dynamic stretching and 17 in the manual therapy group using static stretching, and there was no significant difference between the groups (Table 1).

The study measured changes in pain before and after the intervention within each group using the Numeric Pain Rating Score (NPRS), and the results are presented in Table 2. Regarding the mean difference in NPRS scores before and after the intervention, there was no significant difference between DSMT and SSMT. However, the results showed a significant effect of time (*p* < 0.001) on NPRS.

A goniometer was used to measure changes in the range of motion before and after the intervention within the group (Table 3). The mean difference in the ROM of shoulder flexion and abduction was not significantly different between DSMT and SSMT. However, the results showed a significant effect of time (*p* < 0.001) on shoulder flexion and abduction. There was a significant difference between the two groups before and after the intervention (*p* < 0.05). The results showed a significant interaction between the Time × Group effect, with *F* values of *F* = 4.401, *p* < 0.05 for external rotation and *F* = 9.976, *p* < 0.05 for internal rotation. This means that the treatment effect appears over time.

Changes in function before and after the intervention within the group were measured using the Korean version of the shoulder disability index, and the following results were obtained (Table 4). Regarding the mean difference in shoulder disability index before and after intervention, there was no significant difference between DSMT and SSMT. However, the results showed a significant effect of time (*p* < 0.001) on SPADI-P, SPADI-D, and SPADI-T.

Quality of life was measured before and after the intervention within each group using the SF-36. The results are presented in Table 5. Regarding the mean difference in quality of life before and after intervention, there was no significant difference between DSMT and SSMT in SF-36PCS and SF-36MCS. However, the results showed a significant effect of time (*p* < 0.001). There was a significant difference observed between the two groups before and after the intervention (*p* < 0.05) in SF-36GH. The results showed a significant interaction between the Group × Time effect, with *F* values of *F* = 5.172, *p* < 0.05 for SF-36GH. This means that the treatment effect appears over time.

## 4. Discussion

This study aimed to examine the impacts of dynamic stretching and static stretching combined with manual therapy on pain, range of motion, shoulder function, and quality of life in patients suffering from adhesive capsulitis. The shoulder is a common site of pain in clinical practice, and pain in the shoulder area can be caused not only by issues with the shoulder joint, surrounding ligaments, and muscles, but also by other factors such as neurogenic pain originating from the cervical spine, heart disease, and metastatic pain originating from the diaphragm.

The NPRS (Numeric Pain Rating Score) is used to assess the average level of pain experienced by a patient over the past 24 h, with scores ranging from 0 (indicating no pain) to 10 (indicating unbearable pain) [25]. Stretching techniques are known to alter collagen arrangement to create mechanical effects, stimulate mechanoreceptors, and improve joint pain and patient function [29].

The study found that both dynamic stretching and static stretching interventions resulted in a significant decrease in pain, as measured by the numeric pain rating scale. In the A group, the score decreased from 5.88 to 2.35 (*p* < 0.001), and in the B group, the score decreased from 5.52 to 2.41 (*p* < 0.001). The results were consistent with a previous study by Iqbal et al. [29], on 60 patients with stage 1 and 2 adhesive capsulitis, where there was no significant difference in pain reduction between the A and B groups. Their study measured the two groups exposed to energetic and passive stretching using the numeric pain rating scale, goniometer, shoulder pain, and disability index, and the quick version of the disabilities of the arm, shoulder, and hand questionnaire. These findings suggest that both dynamic and static stretching can effectively lower pain in patients with adhesive capsulitis. In this study, it is believed that both the A group and B group experienced pain relief through myofascial release-based manual therapy applied to shoulder joint-related muscles. In addition, this study did not classify adhesive capsulitis by stage, which may have affected the homogeneity of the test results. Therefore, future studies should subdivide the disease by stage of onset to gain a better understanding of the effectiveness of different interventions.

In this study, both groups showed significant increases in flexion, abduction, internal rotation, and external rotation after intervention. Group A (or DSMT) showed a significant increase in external rotation (ER) from a pre-intervention measurement of 31.0 degrees to a post-intervention measurement of 66.94 degrees (an increase of 33.88 degrees, *p* < 0.000). Internal rotation (IR) also increased from 12.11 degrees to 45.70 degrees (an increase of 33.58 degrees, *p* < 0.000). In Group B (SSMT), ER increased significantly from 32.76 degrees pre-intervention to 62.94 degrees post-intervention (an increase of 30.17 degrees, *p* < 0.000), and IR increased from 13.64 degrees to 37.41 degrees (an increase of 23.76 degrees, *p* < 0.000). Both groups demonstrated significant pre–post differences, and when comparing between groups, Group A (or DSMT) showed a statistically significantly greater increase in ER by 5.69 degrees and IR by 9.82 degrees compared to Group B (SSMT) (*p* < 0.05).

Static stretching involves slow, controlled movements to the endpoint of the range of motion (ROM) before pain occurs, using an external force to gradually stretch the tissues around the shoulder, improving joint coordination to enhance performance [30]. On the other hand, dynamic stretching consists of controlled movements that include active joint movements through a regulated range of motion, achieved through repetitive cyclic muscle loading (tension associated with achieving end ROM) and unloading (muscle relaxation through mid-ROM) using voluntary force [31].

The stiffness of the posterior inferior capsule and rotator cuff is the main cause of the reduction in the range of motion during the medial and external rotation of the Glenohumeral joint. The increased stiffness of the posterior structure causes the humerus head to move anteriorly or posteriorly, resulting in the arm being raised or medially rotated, which may induce pain during rotation or external rotation [32]. Static, dynamic, and proprioceptive neuromuscular facilitation have all been reported to increase the range of motion [33]. Dynamic stretching, in particular, enhances the activation of neuromuscular function, increasing power output [24]. Therefore, it can be inferred that the significant difference in the range of motion between groups for internal and external rotation was due to the reduction of pain and the improvement of force output by activation of the neuromuscular function. Additionally, since there was no difference observed in the range of motion of flexion and abduction between the groups, this may be due to an insufficient number of subjects, and a supplementary study is needed to confirm these results.

Adhesive capsulitis is characterized by pain and stiffness, leading to a gradual reduction in active and passive shoulder joint movements and a secondary reduction in muscle strength and endurance due to disuse [34]. Stiffness in the shoulder joint indicates a progressive loss of function, with a limited range of motion in all shoulder joint movements due to joint capsule contracture. Patients primarily experience difficulty in activities that require bringing their hands back, leading to muscle tension and reduced muscle strength and endurance due to overload, which further limits functional activities and daily living [35].

In this study, the function of patients with adhesive capsulitis was measured using the Korean version of the shoulder disability index pain subscale. The A group showed statistically significant reductions in SPADI-pain, disability, and total scores after the experiment, and the B group also exhibited statistically significant reductions in these scores after the experiment. However, there was no significant difference when comparing the differences between the groups based on the treatment method.

Adhesive capsulitis is a condition that causes pain and functional limitations by reducing the range of motion of the shoulder joint [36]. Stretching techniques are effective in improving muscle elasticity and can improve the passive and active range of motion in patients with adhesive capsulitis [37]. Furthermore, the benefit of dynamic stretching lies in the unloading that occurs in the mid-range of motion, which assists in the relaxation of the muscles around the shoulder joint. At the same time, the loading of the muscles that occurs at the end range of motion effectively enhances the joint range of motion [31]. This suggests that dynamic stretching is more helpful in increasing joint range of motion.

This study found that both the dynamic stretching and static stretching groups showed statistically significant improvements in pain and range of motion. Improvements in pain and range of motion can lead to better shoulder function in patients with adhesive capsulitis. Therefore, dynamic stretching combined with manual therapy is believed to increase muscle temperature and range of motion (ROM), leading to pain relief and improved shoulder function in patients with adhesive capsulitis. Additionally, stiffness in the structures behind the shoulder can lead to anterior–superior migration of the humerus head, scapular protraction, and anterior tilt [38,39]. This migration-related subacromial compression is associated with the restriction of internal rotation range of motion (ROM) [40]. The subscapularis muscle, one of the rotator cuff muscles, is the only muscle among the rotator cuff muscles that generates internal rotation movement at the glenohumeral (GH) joint and contributes to anterior stability, maintaining the proper position of the GH joint to prevent anterior dislocation [41].

Dynamic stretching, involving muscle contractions driven by voluntary force rather than passive static stretching, is believed to enhance the activation of the subscapularis muscle’s neuromuscular activity [31]. This leads to effective internal rotation movement while counteracting the abnormal anterior–superior migration of the humerus head within the GH joint caused by stiffness in the structures behind the shoulder. During dynamic stretching, the activated subscapularis muscle is thought to facilitate more precise glenohumeral joint movement, resulting in a greater range of motion for internal rotation compared to static stretching.

In this study, changes in the quality of life of patients with adhesive capsulitis were assessed using dynamic stretching and static stretching combined with manual therapy. The A group demonstrated statistically significant increases in SF-36 physical factors, the SF-36 mental component, and SF-36 general health factors after the treatment and the B group also showed statistically significant increases in these factors after the treatment. Additionally, the SF-36 general health factor increased significantly more in the A group compared to the B group.

The upper limb plays an essential role in daily activities, and the shoulder joint is the joint with the largest range of motion in the human body. Adhesive capsulitis is a disease that causes severe pain, limitation of range of motion, and even sleep disturbance, which negatively affects the quality of life [42]. The physical health factors of SF-36, including the physical role limitation category, physical function category, and emotional role limitation category, have relatively higher loading than the mental health factors. The vitality category, general health category, and mental health category are loaded with the physical health category in the mental health factors. The primary factors comprising the physical health factors are in four categories: the physical role limitation category, the physical function 1 category, the physical function 2 category, and the emotional role limitation category. It consists of two health categories and a general health category. However, the pain category model is set to affect both the two secondary factors [22].

In this study, both groups that received dynamic stretching and static stretching combined with manual therapy showed significant improvements in pain, range of motion, and function. Improvements in these areas affect the quality of life of patients with adhesive capsulitis. In particular, the combination of dynamic stretching and static stretching, along with myofascial release-based manual therapy applied to both groups, has the potential to enhance muscle function by reducing adhesions and restrictions in the fascia. The group that received static stretching likely performed slow and controlled movements to the end point of their range of motion (ROM) before experiencing pain, using an external force to gradually stretch the tissues around the shoulder. This may have improved joint coordination and, as a result, enhanced performance [30]. On the other hand, the group that received dynamic stretching likely experienced increased controlled movements through an active range of motion due to repetitive cyclic muscle loading and unloading using voluntary force. This could have led to improved and regulated movements [31]. This can lead to better muscle coordination and overall function. By releasing tension in the fascia, myofascial release can enhance blood and lymphatic circulation, aiding in the removal of metabolic waste products and toxins from the muscles. For these reasons, it appears that the SF-36 physical factors improved. Furthermore, dynamic stretching and static stretching combined with manual therapy are relaxing and can promote a sense of well-being. It may help reduce stress and anxiety by releasing physical tension in the body. This is why the SF-36 mental component seems to have improved.

Due to the limitations of this study, it is recommended to conduct further studies with a larger sample size. Additionally, we were unable to control the use of oral medications and the application of analgesia and other adjunctive therapies such as occupational therapy based on the prescribing physician’s discretion. One month, three months, and six months of follow-up assessments were not conducted after the intervention. In this study, it should be noted that not measuring other psychosocial factors such as mood, catastrophizing, and fear avoidance limits the analysis of crucial factors associated with quality of life, pain, and disability. Therefore, it may be worth considering the inclusion of these factors in future research to provide a more comprehensive understanding of the outcomes. Controlling these aspects would provide more accurate data on the effectiveness of stretching interventions.

## 5. Conclusions

It is important to note that although dynamic stretching was found to be effective in this study, it should not be considered a standalone treatment for adhesive capsulitis. It should be used as part of a comprehensive treatment plan that includes other interventions such as manual therapy, exercise therapy, and medication, as recommended by the attending physician or physical therapist. Significant differences were observed in the pain, flexion, and abduction of shoulder ROM, shoulder function, and the physical and mental components of the SF-36 in both groups. The external and internal rotation of shoulder ROM and general health of SF-36 increased significantly more in the A group (DSMT group) compared to the B group (SSMT).

Further research is needed to investigate the long-term effects of dynamic stretching and to develop standardized guidelines for its use in clinical practice. Additionally, future studies should consider a larger sample size and include a more diverse range of patients with different stages and durations of adhesive capsulitis. Overall, the findings of this study suggest that dynamic stretching can be a valuable addition to the treatment options available for patients with adhesive capsulitis.

## Figures and Tables

**Table 1:** Demographic characteristics (*N* = 34).

Characteristics		DSMT (*n* = 17)	SSMT (*n* = 17)	*χ*^2^*/F*(*p*)
Sex	M/F	4/13	5/12	0.151 (0.697)
Age (years)		52.47 ± 4.63	50.59 ± 5.47	1.081 (0.288)
Height (cm)		164.14 ± 4.84	165.76 ± 5.04	−0.520 (0.607)
Weight (kg)		61.88 ± 9.48	65.76 ± 10.01	−1.161 (0.254)
BMI (kg/m^2^)		22.67 ± 2.52	23.84 ± 2.70	−1.302 (0.202)

Values are expressed as the mean ± standard deviation. DSMT: dynamic stretching combined with manual therapy group; SSMT: static stretching combined with manual therapy group.

**Table 2:** Changes in the numerical pain rating scale (*N* = 34).

Parameters		DSMT Group (*n* = 17)	SSMT Group (*n* = 17)	t(*p*)	Time	Group	Time × Group
					*F*(*p*)	*F*(*p*)	*F*(*p*)
NPRS (score)	Pre-test	5.88 ± 1.49	5.52 ± 1.32	0.728 (0.472)	400.596 (0.000)	0.135 (0.715)	1.537 (0.224)
	Pos-ttest	2.35 ± 0.93	2.41 ± 1.22				
	Mean difference	−3.52 ± 0.87	−3.11 ± 1.05	−1.240 (0.224)			
	t(*p*)	16.641 (0.000)	12.199 (0.000)				

DSMT: dynamic stretching combined with manual therapy group; SSMT: static stretching combined with manual therapy group; NPRS: numeric pain rating scale. Data are presented as mean (standard deviation). Level of significance: *p* < 0.05. *F*: Two-way repeated-measure analysis of variance.

**Table 3:** Change in range of motion (*N* = 34).

Parameters		DSMT Group (*n* = 17)	SSMT Group (*n* = 17)	t(*p*)	Time	Group	Time × Group
					*F*(*p*)	*F*(*p*)	*F*(*p*)
Flexion (°)	Pretest	119.52 ± 13.96	118,17 ± 13.47	0.288 (0.776)	449.189 (0.000)	0.018 (0.893)	0.168 (0.685)
	Posttest	161.29 ± 12.98	161.58 ± 10.67				
	Mean difference	41.76 ± 9.58	43.41 ± 13.51	−0.410 (0.685)			
	t(*p*)	−17.971 (0.000)	−13.240 (0.000)				
Abduction (°)	Pretest	99.94 ± 14.00	98.88 ± 12.23	0.235 (0.816)	408.260 (0.000)	0.325 (0.573)	0.420 (0.521)
	Posttest	144.41 ± 14.04	140.58 ± 15.35				
	Mean difference	44.47 ± 10.57	41.70 ± 14.05	0.648 (0.521)			
	t(*p*)	−17.344 (0.000)	−12.237 (0.000)				
External Rotation (°)	Pretest	31.05 ± 7.35	32.76 ± 6.79	−0.707 (0.484)	589.864 (0.000)	0.253 (0.619)	4.401 (0.044)
	Posttest	66.94 ± 9.44	62.94 ± 7.20				
	Mean difference	35.88 ± 7.70	30.17 ± 8.14	2.098 (0.044)			
	t(*p*)	−19.202 (0.000)	−15.268 (0.000)				
Internal Rotation (°)	Pretest	12.11 ± 5.29	13.64 ± 5.52	−0.825 (0.416)	340.040 (0.000)	3.144 (0.086)	9.976 (0.003)
	Posttest	45.70 ± 7.18	37.41 ± 9.79				
	Mean difference	33.58 ± 7.72	23.76 ± 10.23	3.158 (0.003)			
	t(*p*)	−17.934 (0.000)	−9.571 (0.000)				

Data are presented as mean (standard deviation). Level of significance: *p* < 0.05. *F*: Two-way repeated-measure analysis of variance. DSMT: dynamic stretching combined with manual therapy group; SSMT: static stretching combined with manual therapy group.

**Table 4:** Changes in the Korean version of the shoulder disability index (*N* = 34).

Parameters		DSMT Group (*n* = 17)	SSMT Group (*n* = 17)	t(*p*)	Time	Group	Time × Group
					*F*(*p*)	*F*(*p*)	*F*(*p*)
SPADI-P (%)	Pretest	52.82 ± 10.12	53.29 ± 9.56	−0.825 (0.416)	587.651 (0.000)	0.069 (0.794)	0.015 (0.905)
	Posttest	17.17 ± 5.79	18.00 ± 7.10				
	Mean difference	−35.64 ± 7.97	−35.29 ± 9.05	−0.121 (0.905)			
	t(*p*)	18.427 (0.000)	16.073 (0.000)				
SPADI-D (%)	Pretest	67.27 ± 8.45	67.72 ± 10.04	−0.139 (0.890)	718.980 (0.000)	0.008 (0.927)	0.023 (0.879)
	Posttest	35.22 ± 7.58	35.29 ± 9.22				
	Mean difference	−32.05 ± 5.60	−32.42 ± 8.17	0.153 (0.879)			
	t(*p*)	23.576 (0.000)	16.347 (0.000)				
SPADI-T (%)	Pretest	62.16 ± 3.83	62.16 ± 5.42	0.000 (1.000)	306.650 (0.000)	0.043 (0.837)	0.236 (0.631)
	Posttest	28.27 ± 3.41	28.86 ± 5.05				
	Mean difference	−33.89 ± 2.81	−33.30 ± 4.13	−0.485 (0.631)			
	t(*p*)	49.562 (0.000)	33.225 (0.000)				

SPADI: shoulder pain and disability index; SPADI-P: SPADI-pain; SPADI-D: SPADI-disability; SPADI-T: SPADI-total. Data are presented as mean (standard deviation). Level of significance: *p* < 0.05. *F*: Two-way repeated-measure analysis of variance. DSMT: dynamic stretching combined with manual therapy group; SSMT: static stretching combined with manual therapy group

**Table 5:** Changes in SF-36 (*N* = 34).

Parameters		DSMT Group (*n* = 17)	SSMT Group (*n* = 17)	t(*p*)	Time	Group	Time × Group
					*F*(*p*)	*F*(*p*)	*F*(*p*)
SF-36 PCS (score)	Pretest	59.03 ± 4.94	58.34 ± 4.49	0.424 (0.675)	306.847 (0.000)	0.652 (0.425)	0.231 (0.634)
	Posttest	71.82 ± 6.68	71.85 ± 6.14				
	Mean difference	12.79 ± 4.47	13.51 ± 4.28	−0.480 (0.634)			
	t(*p*)	−11.795 (0.000)	−13.009 (0.000)				
SF-36 MCS (score)	Pretest	57.91 ± 5.67	56.84 ± 3.34	0.671 (0.507)	295.100 (0.000)	0.449 (0.508)	0.157 (0.695)
	Posttest	73.04 ± 8.37	71.29 ± 7.91				
	Mean difference	15.13 ± 4.85	14.45 ± 5.18	0.396 (0.695)			
	t(*p*)	−12.860 (0.000)	−11.493 (0.000)				
SF-36 GH (%)	Pretest	49.11 ± 9.87	49.41 ± 9.66	−0.088 (0.931)	530.182 (0.000)	0.541 (0.467)	5.172 (0.030)
	Posttest	75.29 ± 7.17	70.88 ± 6.61				
	Mean difference	26.17 ± 6.50	21.47 ± 7.75	2.274 (0.030)			
	t(*p*)	−16.599 (0.000)	−16.026 (0.000)				

SF-36: short form 36; SF-36 PCS: SF-36 physical component score; SF-36 MCS: SF-36 mental component score; SF-36 GH: SF-36 general health. Data are presented as mean (standard deviation). Level of significance: *p* < 0.05. *F*: Two-way repeated-measure analysis of variance.

## Data Availability

Data are contained within the article.
